# Protective effects of circulating microvesicles derived from myocardial ischemic rats on apoptosis of cardiomyocytes in myocardial ischemia/reperfusion injury

**DOI:** 10.18632/oncotarget.17424

**Published:** 2017-04-26

**Authors:** Yao Wang, Su Wei, Yi-Lu Wang, Miao Liu, Man Shang, Qi Zhang, Yan-Na Wu, Ming-Lin Liu, Jun-Qiu Song, Yan-Xia Liu

**Affiliations:** ^1^ Department of Pharmacology, School of Basic Medical Sciences, Tianjin Medical University, Tianjin 300070, China; ^2^ Section of Endocrinology, Department of Medicine, Temple University School of Medicine, Philadelphia, PA19140, USA; ^3^ Section of Endocrinology, Department of Medicine, Temple University School of Medicine, Philadelphia, PA 19140, USA; ^4^ Department of Dermatology, Perelman School of Medicine, University of Pennsylvania, Philadelphia, PA 19104, USA

**Keywords:** microvesicles, ischemia/reperfusion, apoptosis, Bcl-2/Bax, GRP78

## Abstract

**Objective:**

To investigate the effects of circulating microvesicles derived from myocardial ischemia (I-MVs) on apoptosis in myocardial ischemia/reperfusion (I/R) injury in rats.

**Methods:**

I-MVs from rats undergoing myocardial left anterior descending (LAD) coronary artery ligation were isolated by ultracentrifugation from circulating blood and characterized by flow cytometry. I-MVs were administered intravenously (4.8 mg/kg) at 5 min before reperfusion procedure in I/R injury model which was induced by 30-min of ischemia and 120-min of reperfusion of LAD in rats.

**Results:**

Treatment with I-MVssignificantly reduced the size of myocardial infarction, the activities of serum CK-MB and LDH, and the number of apoptotic cardiomyocytes. The activities of caspase 3, caspase 9 and caspase 12 in myocardium were also decreased significantly with I-MVs treatment. Moreover, the expression of Bax was decreased but Bcl-2 was increased. The expression of glucose regulated protein 78 (GRP78), sarco/endoplasmic reticulum Ca^2+^-ATPase 2 (SERCA2) and phosphorylated phospholamban (p-PLB) were increased after being treated with I-MVs.

**Conclusion:**

I-MVs could protect hearts from I/R injury in rats through SERCA2 and p-PLB of calcium regulatory proteins to alleviate intrinsic myocardial apoptosis including mitochondrial and endoplasmic reticulum pathways.

## INTRODUCTION

Microvesicles (MVs) are small vesicles of 0.1˜1μm in diameter and deriving from the shedding of cellular membrane during activation or apoptosis. MVs are involved in many physiological processes and increased in a variety of cardiovascular disorders [1]. MVs shed by different cells contain a series of cell surface biomarkers derived from the plasma membrane and a rich source of cellular contents of the parental cells. Thus MVs appear to serve as both markers and mediators to take part in many biological processes [2, 3].

Recent studies indicated that patients with cardiovascular diseases such as ischemia heart disease, atherosclerosis, hypertension have increased circulating levels of MVs [4]. This suggested that under pathological states, MVs may play a part in these processes. Researches demonstrated that higher MV levels were observed in myocardial infarction (MI), especially endothelial microvesicles (EMVs) and platelet microvesicles (PMVs) [5, 6]. Boulanger et al. reported that circulating MVs from patients with MI selectively impaired rat aortic rings, which could contribute to the general vasomotor dysfunction and endothelial apoptosis. Recent evidence suggested that MVs released from the heart after ischemic preconditioning were necessary for cardioprotection during I/R injury in Langendorff heart model of rats [7]. Jeanneteau et al. showed MVs from rats and healthy humans undergoing remote ischemic conditioning (RCond) also increased in circulating levels of EMVs and PMVs. However, injecting RCond-derived MVs to I/R injury rats before reperfusion did not induce cardioprotection [8]. How about I-MVs? So it is of important realistic meaning to research the effects of MVs derived from ischemic myocardium especially on apoptosis in myocardial I/R injury.

Circulating MVs derived from rats of myocardial ischemia should be associated with apoptosis in myocardial I/R injury. In order to test this hypothesis, we purified, characterized, and quantified I-MVs, demonstrating protection against I/R injury in rats. We speculated that this cardioprotection was mediated by Bcl-2/Bax in mitochondrial pathway and/or GRP78 in endoplasmic reticulum pathway which was regulated by a common upstream of SERCA2 and p-PLB of calcium regulatory proteins.

## RESULTS

### Hemodynamic and electrophysiological changes

I/R injury model *in vivo* in rats was established successfully according to the changes of HR, MAP and ST-T segment throughout the whole process of experiment and VA during ischemia period. During ischemia, HR was decreased, MAP was dropped and ST-segment was elevated dramatically. HR was increased, MAP was elevated and ST-segment was fallen after reperfusion. All kinds of VA appeared in ischemia period including ventricular premature contraction, ventricular tachycardia and ventricular fibrillation (Data not shown).

### Isolation and characterization of I-MVs

**Figure 1:**
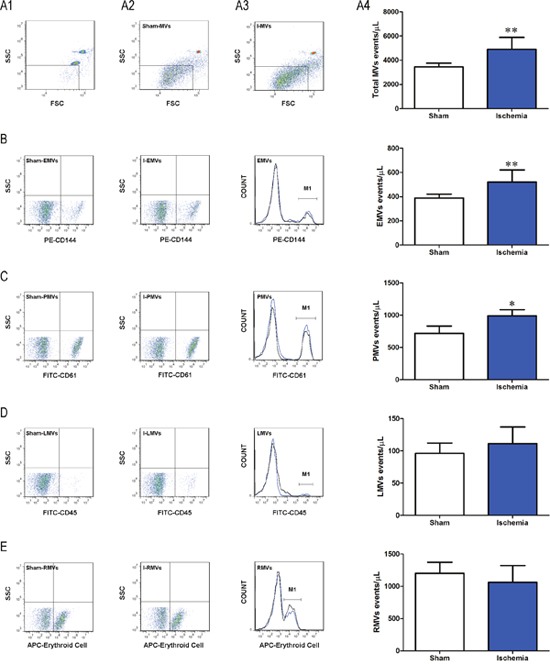
Characterization of I-MVs by flow cytometry **(A1)** Representative dot plot of FSC *vs* SSC for evaluation of background noise in the sample of purified water. **(A2-3)** Representative dot plots for total MVs from sham and ischemia groups, respectively. **(A4)** Summary and statistical analysis of total MVs in these two groups. **(B, C, D)** and **(E)** Display the representative dot plots, histograms and total numbers of MVs derived from endothelium (B, anti-CD144-PE), platelets (C, anti-CD61-FITC), leukocytes (D, anti-CD45-FITC) and erythrocytes (E, anti-erythroid cell-APC) in ischemia versus sham groups, respectively. In histograms, black line and blue line represent sham-MVs and I-MVs, respectively. M1 means events of positive MVs of different cell types in each group (B-E). Values are mean±SD, n=8, *P<0.05, **P<0.01 *vs* sham.

After two sequential centrifugations, cells and cell debris were eliminated. It was observed that events were mainly located under the region of 1-μm beads (Figure 1A1-1A4). In contrast, we found that the large amount of MVs located in the area smaller than 1 μm which were confirmed by 1-μm standard beads. With the known number of 2-μm beads added, the number of MVs in sham and ischemia groups was calculated. The total number of circulating I-MVs was significantly increased compared with sham group (4,897±996 *vs* 3,453±307 events/μL of plasma, *P*<0.01).

Phenotypical characterization of the cellular origin of I-MVs showed a significant increase in EMVs (CD144+) and PMVs (CD61+) in the 30 min ischemia versus sham group (521±99 *vs* 388±33, *P*<0.01, 990±96 *vs* 718±114, *P*<0.05 events/μL of plasma, respectively) (Figure 1B and 1C). No significant differences were observed for I-MVs from other cellular regions including LMVs (CD45+) and RMVs (APC-Erythroid cell+) (Figure 1D and 1E).

### Tissue damage in I/R rats after treatment of I-MVs

**Figure 2:**
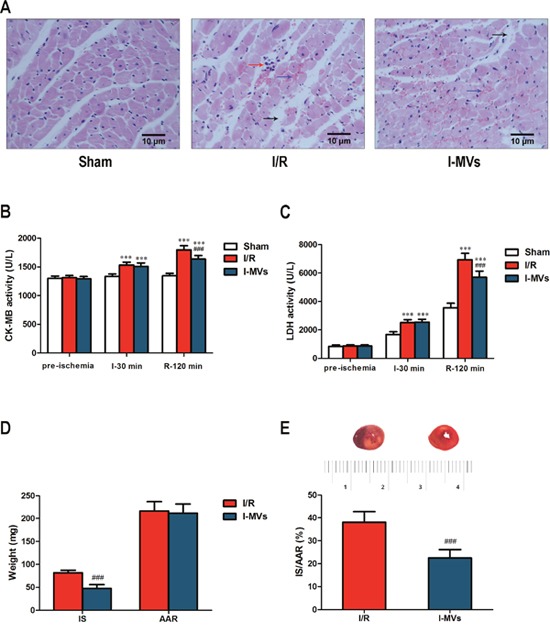
Effects of I-MVs on myocardial I/R injury **(A)** HE staining of myocardium at the end of 120 min reperfusion. The arrows show different tissue damages, such as interstitial edema (black arrow), erythrocyte extravasation (blue arrow) and leukocyte infiltration (red arrow). **(B)** and **(C)** CK-MB and LDH activities of serum among pre-ischemia, ischemia 30 min (I-30 min) and reperfusion 120 min (R-120 min). **(D)** Cardiac tissue weight of IS and AAR. **(E)** Myocardial infarct size expressed as ratio of IS to AAR (IS/AAR %) and the representative photographs of myocardial tissue sections in each group. Values are mean±SD, n=8, ***P<0.001 *vs* sham, ^###^*P*<0.001 *vs* I/R.

In the HE staining assay, sham was shown no difference compared to normal tissue. Necrotic cells with swelling, karyolysis, cytoplasmic uniformity, disintegration, erythrocyte extravasation and neutrophil infiltrating were found in the ischemia area in other two groups. Morphologic changes of cardiomyocytes in I-MVs group were obviously alleviated compared with I/R group (Figure 2A).

CK-MB activity remained steady in sham group during I/R. However activities increased significantly for other two groups at the end of ischemia and after 120 min reperfusion, especially in latter time point. Compared with I/R, CK-MB activity in I-MVs significantly decreased after 120 min reperfusion (1,637±61 *vs* 1,797±73 U/L, *P*<0.001) (Figure 2B).

LDH activity gradually increased in sham group during I/R. Compared with sham group, LDH activities were distinctly increased at the end of 30 min ischemia and 120 min reperfusion both in I/R and I-MVs. Compared with I/R, the activity of LDH was significantly decreased after 120 min reperfusion (5,706±432 *vs* 6,933±460) U/L, *P*<0.001) (Figure 2C).

TTC staining showed that AAR was similar, however, IS was significantly different in I/R and I-MVs (81.78±5.45 *vs* 47.61±8.36 mg, *P*<0.001). The ratio of IS to AAR was significantly lower in I-MVs than I/R (22.49±3.66 *vs* 38.01±4.74 %, *P*<0.001) (Figure 2D and 2E).

### Effects of I-MVs on myocardial apoptosis in I/R injuried rats

**Figure 3:**
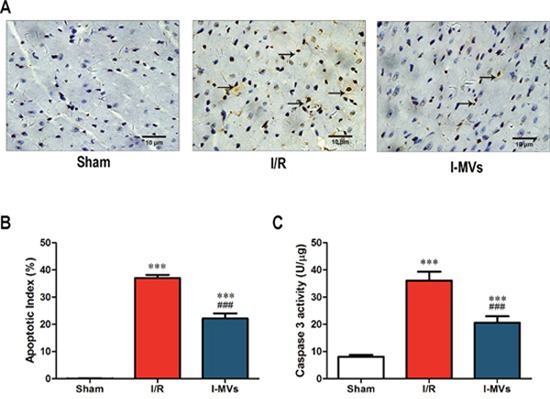
Effects of I-MVs on myocardial apoptosis **(A)** Myocardial apoptosis detected by TUNEL staining. TUNEL-positive nuclei are stained brown (indicated by arrows) and TUNEL-negative nuclei stained blue. **(B)** Counting and analysis of total and TUNEL-positive nuclei in these three groups. AI indicates percentages of TUNEL-positive nuclei in cardiac tissue sections. **(C)** Activities of caspase 3 in myocardium. Values are mean±SD, n=8, ***P<0.001 *vs* sham, ^###^*P*<0.001 *vs* I/R.

In the TUNEL staining assay, TUNEL-positive nuclei, which were apoptotic cells’ nuclei stained brown and revealed condensation of chromatin, were found in the ischemic area. There were little TUNEL-positive nuclei in sham group. In other two groups, there were more TUNEL-positive nuclei. In contrast to I/R, the percentage of TUNEL-positive nuclei in I-MVs obviously decreased (Figure 3A). Ten fields were examined in each slice and the counts were averaged. AI was significantly reduced in I-MVs, compared with I/R (22.14±1.89 *vs* 37.03±1.18 %, *P*<0.001) (Figure 3B).

**Figure 4:**
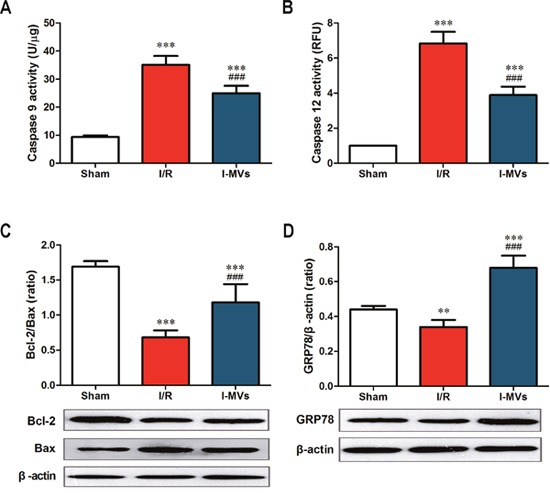
Effects of I-MVs on myocardial apoptosis pathways **(A)** and **(B)** Activities of caspase 9 and 12 in myocardium (n=8). **(C)** and **(D)** Summary analyses and their corresponding representative immunoblotting of Bcl-2, Bax, and GRP78 (n=6). Values are mean±SD, **P<0.01, ***P<0.001 *vs* sham, ^###^*P*<0.001 *vs* I/R.

The caspase 3 activity of the risk area in myocardium in I/R and I-MVs was significantly risen compared with sham (*P*<0.001). However, in contrast to I/R, the caspase 3 activity in I-MVs was significantly fallen (20.59±2.38 *vs* 36.10±3.23 U/μg, *P*<0.001). The changes of caspase 9 and 12 activitied were similar to caspase 3 (25.00±2.64 *vs* 35.10±3.15 U/μg, 5.54±0.80 *vs* 9.84±1.02 RFU/mg, respectively, *P*<0.001) (Figure 3C, Figure 4A and 4B).

The expressions of Bcl-2, Bax and GRP78 were determined by Western blotting analysis. Results indicated that the expression of Bax was markedly higher but Bcl-2 lower in other two groups especially in I/R. Compared with I/R, I-MVs decreased the expression of Bax and increased that of Bcl-2, so the ratio of Bcl-2/Bax was clearly increased in I-MVs (1.18±0.26 *vs* 0.68 ±0.10 %, P<0.001) (Figure 4C). The expression of GRP78 was clearly decreased in I/R, nevertheless, significantly increased in I-MVs group. So compared with I/R, the expression of GRP78 was evidently increased in I-MVs (0.68±0.07 *vs* 0.34 ±0.04, P<0.001) (Figure 4D).

### Effects of I-MVs on SERCA2 and p-PLB in I/R injuried rats

**Figure 5:**
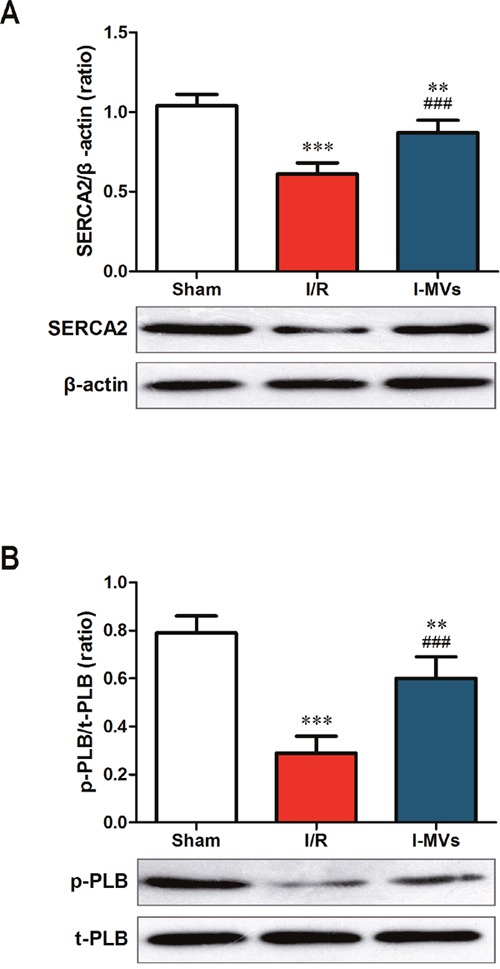
Effects of I-MVs on calcium relative regulated proteins **(A)** and **(B)** Summary analyses and their corresponding representative immunoblotting of SERCA2 (A), p-PLB and t-PLB (B), respectively. Values are mean±SD, n=6, **P<0.01, ***P<0.001 *vs* sham, ^###^*P*<0.001 *vs* I/R.

SERCA2, a kind of Ca^2+^ ATPase that transports Ca^2+^ from the cytoplasm of the cell to the lumen of the ER, is an important regulator of Ca^2+^ in the cells. Endogenous p-PLB, is an active form of regulatory protein for SERC2 activity. Results indicated that the expressions of SERCA2 and p-PLB were lower in other two groups especially in I/R, in contrast to sham group. As a result, compared with I/R group, the expression of SERCA2 and ratio of p-PLB/PLB were markedly increased in I-MVs (0.87±0.08 *vs* 0.61 ±0.07, 0.60±0.09 *vs* 0.29 ±0.07, respectively, P<0.001) (Figure 5).

## DISCUSSION

Circulating MVs have emerged as a new concept biomarker in ischemic heart disease. The number and source of MVs in ischemic heart diseases may be changed. Elevated levels of circulating MVs had been tested in patients with ST elevation myocardial infarction (STEMI), especially EMVs and PMVs [13]. Another study reported that the levels of MVs in acute myocardial infarction (AMI) were higher than stable angina, which included EMVs, PMVs and monocytes derived MVs [5, 6, 14]. Similar results in rats have been observed in this study. After 30 min ligation of LAD coronary artery, levels of total MVs, EMVs and PMVs were significantly elevated with myocardial ischemia rats as compared to control group, while LMVs and RMVs were not obviously different. These results indicate that a direct relationship removed between the ischemic stimulus and the varieties of count and origin in circulating levels of MVs. The varieties prompt that MVs from the ischemic conditions play a significant role in the development and progression of myocardial ischemia. So our research can imitate varieties of circulating MVs in patients of MI and explore their effects, and that provide necessary basis of experiment for clinical prevention, diagnosis and therapy of ischemic heart disease. MVs generated in normal physiological condition were few. Therefore, I-MVs were used for functional studies.

In addition, many faces of circulating MVs are closely associated with which role may be play. Several studies had suggested that the release of MVs was a protective mechanism to clear cell stress in the patients with coronary diseases [15–18]. Meanwhile, some studies indicated that MVs played an important role in tissue regeneration repair and protection, because they were strongly associated with angiogenesis [19–22]. Our previous studies demonstrated that I-MVs alleviated apoptosis of cultured H9c2 cardiomyocytes which were treated by hypoxia/reoxygenation and MVs released from human umbilical vein endothelial cells exerted a pro-apoptotic effect on cultured H9c2 cardiomyocytes. Our present study identified that activities of serum CK-MB and LDH were obviously decreased after treatment of I-MVs. The damage and necrosis of myocardial tissue was evidently mitigated through HE staining observation. TTC staining found myocardial infarction size was significantly shrank. Furthermore, TUNEL staining displayed the percentage/number of TUNEL-positive nuclei was clearly decreased, and the activity of caspase 3 in myocardium was remarkably decreased after treatment of I-MVs. The results indicated I-MVs can attenuate tissue death degree and then protect the structure and function of heart through controlling apoptosis of myocardium. In order to research the mechanism of protection, we choose the mitochondrial apoptosis pathway for further study.

Intrinsic apoptosis pathways including mitochondrial and endoplasmic reticulum pathways play a critical role in the pathogenesis of myocardial I/R injury. On the one hand, obstruction of energy production and destruction of calcium homeostasis contribute to mitochondrial dysfunction and initial apoptosis during I/R injury of heart. The Bcl-2 family proteins play a central role in regulating mitochondrial apoptosis [23]. Our results showed the activity of caspase 9 was remarkably decreased in myocardium undergoing I/R injury after treatment with I-MVs. At the same time, Western blotting showed that the expression of Bax was fallen, yet the Bcl-2 rose by using I-MVs treatment. On the other hand, calcium homeostasis is critical in maintaining normal ER function. ER stress refers to a condition in which normal ER function is impaired, leading to accumulation of unfolded or mis-folded proteins in the ER lumen in myocardial I/R injury. In the case of severe or prolonged ER stress, its responses exceed handling ability of GRP 78 and result in the activation of ER stress related apoptosis signaling pathways [24]. Caspase 12 is required for apoptosis induced specifically by ER stress. Our results showed that the activity of caspase 12 was evidently decreased in myocardium by using I-MVs treatment. Meanwhile, Western blotting showed that the expression of GRP 78 was risen after treatment with I-MVs. The results point out I-MVs may attenuate myocardial apoptosis via influencing intrinsic apoptosis pathway.

Calcium and its regulatory proteins play an important role in the development and progression of myocardial apoptosis. Moreover, Ca^2+^ homeostasis is critical in maintaining normal mitochondria and ER function. It is well established that myocardial I/R injury is accompanied by intracellular Ca^2+^ overload which contributes to myocardial apoptosis and death. The depression of Ca^2+^ concentration in cardiomyocytes mostly depends on SERCA2. If the expression of SERCA2 is fallen and function impaired in myocardial apoptosis, a large number of Ca^2+^ are detained in the cytoplasm [25–27]. A decrease in p-PLB level has been considered to reduce SERCA2 activity in the course of I/R injury in the hearts. Our study demonstrated that the expression of SERCA2 and the level of p-PLB were increased by using I-MVs treatment in myocardial I/R injury in rats. It revealed that myocardial apoptosis especially intrinsic apoptosis pathway could be alleviated via increased SERCA2 expression as well as enhanced PLB phosphorylation levels.

Circulating MVs represented transport vehicles for large numbers of specific protein, messenger RNAs, and micro RNAs which have been associated with cardiovascular diseases, and then altered the gene expression, proliferation, and differentiation of the recipient cells [28, 29]. Studies suggested that PMVs contained caspase 9 and procaspase 3 but not caspase 8, and the time course of caspase 9 formation paralleled with procaspase 3 disappearance and caspase 3 appearance. Incubation of human macrophages with PMP induced apoptosis [30]. Schock et al. found that MVs contained caspase 3 and treating the MVs with a caspase 3 inhibitor significantly reduced cell apoptosis [1]. Another results represented that coronary heart disease was associated with changes in the transport of circulating miRNA, particularly decreased miRNA enrichment in MVs [31]. These findings described an interesting packaging mechanism for transferring information function from MV-releasing cells to target cells via MVs circulating in blood. Our results showed the activities of caspase 3, 9 and 12, the expression of Bax in myocardium were decreased, while the expressions of Bcl-2, GRP78 and SERCA2 and the ratio of Bcl-2/Bax and p-PLB/PLB were increased by using I-MVs treatment. Therefore we speculated I-MVs may serve as a vehicle to transfer some kinds of caspases and apoptosis related proteins to reduce target cell apoptosis through intrinsic apoptosis pathway in myocardial I/R injury. Whether or not needs study further.

In summary, our findings have demonstrated that circulating I-MVs could reduce myocardial apoptosis of I/R injuried rats. Moreover, one of the underlying mechanisms is that I-MVs exert an anti-apoptotic effect on myocardium and protection of heart *in vivo* through affecting calcium regulatory proteins and intrinsic apoptosis pathway. Further studies should be carried out to clarify which kind of I-MVs is able to reduce myocardial apoptosis such as PMVs and EMVs during I/R injury and identify the contents of I-MVs by the means of proteomics or microarray to explore more underlying mechanisms. As both biomarkers and mediators, I-MVs will become increasingly valuable for risk prediction, diagnosis and therapy on ischemic heart disease and potential targets for therapy.

## MATERIALS AND METHODS

### Materials

Male Wistar rats aged 8-10 weeks and weighing 230±10 g were purchased from Academy of Military Medical Sciences. This investigation comformed to the Guide for the Care and Use of Laboratory Animals published by the US National Institutes of Health (NIH Publication No. 85-23, revised 1996) and was approved by the animal ethics committee of Tianjin Medical University, Tianjin, China.

Anti-PE-CD144, anti-Bcl-2 and anti-p-PLB antibodies were from Santa Cruz, anti-FITC-CD61, anti-FITC-CD45, anti-APC-Erythroid Cells antibodies, and their relevant isotypes were from BD Biosciences. Flow Cytometry Size Calibration Kit was from Molecular Probe. TTC was from Sigma and TUNEL Kit was from Roche. CK-MB Kit was from Kewo Biological Technology and LDH assay Kit was from JianCheng Bioengineering Institute. Caspase 3 and 9 assay Kits were from Beyotime Institute of Biotechnology. Caspase 12 assay Kit was from Biovision. Anti-Bax, anti-GRP78, anti-SERCA2 and anti-PLB antibodies were from Cell Signaling Technology.

### Preparation of circulating I-MVs and protein quantification

Myocardial ischemic model of rats was established. Rats were anesthetized with an intraperitoneal injection of urethane (1 g/kg) and ventilated using a small animal ventilator at the rate of 50-60 breaths per minute. After tracheotomy, positive-pressure artificial respiration was started immediately with room air. After the pericardium was incided, a 3-0 silk suture was quickly placed under the LAD coronary artery. A small vinyl tube was threaded through the ligature and placed in contact with the heart. Applying tension to the ligature could occlude the LAD for 30 min.

Circulating I-MVs were isolated. At the end of 30 min of ischemia, blood was collected in corresponding 3.5% sodium citrate tubes after abdominal aorta puncture. Samples were centrifuged for 15 min at 2, 600 g at room temperature, and then plasma was centrifuged for 5 min at 10, 000 g at 4 °C to obtain platelet-free plasma (PFP). PFP in aliquots of 90 μL was fixed with paraformaldehyde (PFA) to a final concentration of 1% for 1 h at room temperature, snap-frozen in liquid nitrogen and stored at -80°C until analysis. The remaining PFP was ultracentrifuged at 100, 000 g for 1 h, and supernatant was replaced by 0.9% sodium chloride, then stored at -80°C until subsequent use [9].

Total rat blood volume was estimated using the formula [0.06×body wt (g) +0.77] [8]. The volume of blood sample, which was a quarter of total volume, was calculated according to the body weight of each rat. I-MVs from each rat were mixed. On the basis of protein quantification, the dosage of I-MVs was then calculated according to the mean circulating level of MVs detected in the rat plasma. Protein quantification of I-MVs was performed by BCA protein assay.

### Flow cytometric analysis of I-MVs

Fixed PFP was blocked with mouse serum subsequently and then incubated with antibody and its isotype for 30 min at room temperature in the dark, respectively. These include anti-PE-CD144 and anti-PE Mouse IgG1 isotype, anti-FITC-CD61 and anti-FITC Mouse IgG1 isotype, anti-FITC-CD45 and anti- FITC Mouse IgG1 isotype, anti-APC-Erythroid Cells and anti-APC Mouse IgM isotype. After being diluted in 300 μL PBS, 50, 000 calibration beads with the diameter of 2 μm were added immediately by Accuri C6 flow cytometer before analysis. Dot plots of forward scatter (FSC) *vs*. side scatter (SSC) were established. Gain setting was adjusted by placing 1-μm beads in the upper log for scatter. Events <1 μm diameter were considered as total I-MVs from circulating blood. Among total MVs, PE-CD144, FITC-CD61, FICT-CD45 and APC-Erythroid Cells positive events were defined as EMVs, PMVs, leukocyte MVs (LMVs) and erythrocyte MVs (RMVs), respectively.

### Treatment of I-MVs on I/R injuried rats

Myocardial I/R injury model of rat was established [10]. Tension was exerted to the ligature temporarily 30 min occluding the LAD and reperfusion was achieved by releasing it for 120 min. Lead II of the electrocardiogram (ECG) for each rat was continuously recorded with the BL-420E biology functional experimental system. ECG changes including variation of ST- segment and ventricular arrhythmia (VA), mean arterial pressure (MAP) and heart rate (HR) were measured.

Rats were randomly assigned to one of the following groups: (1) sham (n=8), rats underwent thoracotomy and the suture threaded but not ligated; (2) I/R (n=16), rats received I/R process; (3) I-MVs (n=16), I-MVs (4.8 mg/kg) were infused via the femoral vein in I/R injury rats. The same volume of 0.9% sodium chloride was given to other two groups. All treatments began at 25-min ischemia, with additional 1 min infusion [11, 12].

### Observation of myocardial morphology

After 120 min reperfusion, the hearts were rapidly removed. The samples obtained from apex of left ventricle were fixed with 10% formalin for 24 h, and then embedded in paraffin and sliced. Sections in 5 μm thick were stained by HE staining. These slices were observed under microscope with magnification of 400×.

The TUNEL protocol was based on TUNEL detection Kit. Pretreatment of myocardium was the same as HE staining. The fixed tissues were embedded in paraffin and 5-μm thick sections were deparaffinized by washing in xylene and a descending ethanol series. The sections were subsequently incubated with 20 μg/mL proteinase K for 30 min at 37°C, and endogenous peroxidase was inactivated by 3% H_2_O_2_ in methanol for 10 min. They were incubated with 50 μL of TUNEL reaction mixture on the section for 60 min at 37°C, and then added 50 μL converter-POD on sample for 30 min at 37°C. For the color development, sections were added with 50 μL DAB substrate for 10 min at room temperature to detect labeled nuclei, and then counterstained by hematoxylin. For each slide, 10 separate fields were examined randomly and digitized by microscopy at magnification of 400×. The apoptotic index (AI) was calculated as the ratio of TUNEL-positive cells to total number of myocytes.

### CK-MB and LDH activity assay

Samples of venous blood in 0.6 mL were collected after anesthesia, 30 min ischemia and 120 min reperfusion, and centrifuged at 2, 000g for 20 min. The CK-MB and LDH in serum were measured using a Microplate Reader, at wavelength of 450 nm.

### Measurement of infarct size of hearts

After 120 min reperfusion, the LAD was religated at its original site. 2 mL of 0.6% Trypan blue was injected into the femoral vein to define the area at risk (AAR). The heart was removed immediately and placed in refrigerator at -20°C for 20 min. Then the left ventricle was sliced into 1 mm slices transversely from apex to base. The slices were incubated in 1% TTC in phosphate buffer solution, pH 7.4, at 37°C for 20 min and then fixed in 10% formalin for 24 h. For each section, AAR (red area) and infarct size (IS, white area) were separated and weighed, respectively. IS was expressed as a percentage of AAR.

### Caspases activities assay

Caspase 3, 9 and 12 activities detection were performed using Caspase 3, 9 and 12 Activity assay Kits. After the hearts were harvested and flushed free of blood, specimens of the front wall of left ventricle (LVFW) were frozen in liquid nitrogen and stored at -80°C until use. These tissues were homogenized in cold lysis buffer for 10 min and centrifuged at 16, 000 g for 20 min. About 20 μg protein extract was incubated in reaction buffer containing 2 mM Ac-DEVD-pNA (caspase 3 substrate) or 2 mM Ac-LEHD-pNA (caspase 9 substrate) for 2 h at 37°C and then measured by a Multilabel Reader at 405 nm. About 150 μg protein extract was incubated in reaction buffer containing 50 μM ATAD-AFC (caspase 12 substrate) for 1 h at 37°C and then measured by fluorescence microplate.

### Western blotting for detecting proteins

Pretreatment of myocardium was the same as caspase activity assay. From -80°C surrounding, the LVFW was homogenized in cold cell lysis buffer and centrifuged at 10, 000 g for 5 min. Samples were heated before electrophoresis (5 min, 100°C). Proteins were separated by 12% Tris/glycine SDS-polyacrylamide gel and transferred to a nitrocellulose membrane which was blocked for 1 h at room temperature and incubated overnight at 4°C with appropriate primary antibodies, including anti-Bcl-2 (1:500), anti-Bax (1:1000), anti-GRP78 (1:1000), anti-SERCA2 (1:1000), anti-PLB (1:1000) and anti-p-PLB (1:500) antibody. Excess primary antibodies were washed from nitrocellulose membrane with three 10-minute washed in TBS-T, and then, these membranes were incubated with goat anti-rabbit polyclonal secondary antibody diluted 1:5000 in blocking buffer. After 3 times of 10-minute washed in TBS-T, attached antibodies were detected with the enhanced chemiluminescence Kit and exposed to Kodak X-omat AR film. Autoradiograph was scanned and relative density quantified.

### Statistical analysis

Results were expressed as mean ±standard deviation (SD). The student t test was performed to compare individual data sets between two groups, which the differences among the groups were tested for significance by one-way ANOVA using Bonferroni's correction for multiple comparisons. *P* value <0.05 was considered as statistically significant. Statistical evaluation was performed by using SPSS 17.0 software.
